# Integration of multimodal imaging data with machine learning for improved diagnosis and prognosis in neuroimaging

**DOI:** 10.3389/fnhum.2025.1552178

**Published:** 2025-03-21

**Authors:** Saurabh Bhattacharya, Sashikanta Prusty, Sanjay P. Pande, Monali Gulhane, Santosh H. Lavate, Nitin Rakesh, Saravanan Veerasamy

**Affiliations:** ^1^School of Computer Science and Engineering, Galgotias University, Greater Noida, Uttar Pradesh, India; ^2^Department of Computer Science and Engineering, ITER-FET, Siksha ‘O’ Anusandhan (Deemed to be University), Bhubaneswar, Odisha, India; ^3^Department of Computer Technology, Yeshwantrao Chavan College of Engineering, Nagpur, Maharashtra, India; ^4^Symbiosis Institute of Technology, Nagpur Campus, Symbiosis International (Deemed University), Pune, India; ^5^Department of Electronics and Telecommunication Engineering, AISSMS College of Engineering, Pune, Maharashtra, India; ^6^Department of Computer Science, College of Engineering and Technology, Dambi Dollo University, Dambi Dollo, Oromia, Ethiopia

**Keywords:** multimodal imaging, structural MRI (sMRI), functional MRI (fMRI), neurological disorders, deep learning framework, data fusion, diagnosis and prognosis

## Abstract

**Introduction:**

Combining many types of imaging data—especially structural MRI (sMRI) and functional MRI (fMRI)—may greatly assist in the diagnosis and treatment of brain disorders like Alzheimer’s. Current approaches are less helpful for forecasting, however, as they do not always blend spatial and temporal patterns from different sources properly. This work presents a novel mixed deep learning (DL) method combining data from many sources using CNN, GRU, and attention techniques. This work introduces a novel hybrid deep learning method combining CNN, GRU, and a Dynamic Cross-Modality Attention Module to help more efficiently blend spatial and temporal brain data. Through working around issues with current multimodal fusion techniques, our approach increases the accuracy and readability of diagnoses.

**Methods:**

Utilizing CNNs and models of temporal dynamics from fMRI connection measures utilizing GRUs, the proposed approach extracts spatial characteristics from sMRI. Strong multimodal integration is made possible by including an attention mechanism to give diagnostically important features top priority. Training and evaluation of the model took place using the Human Connectome Project (HCP) dataset including behavioral data, fMRI, and sMRI. Measures include accuracy, recall, precision and F1-score used to evaluate performance.

**Results:**

It was correct 96.79% of the time using the combined structure. Regarding the identification of brain disorders, the proposed model was more successful than existing ones.

**Discussion:**

These findings indicate that the hybrid strategy makes sense for using complimentary information from several kinds of photos. Attention to detail helped one choose which aspects to concentrate on, thereby enhancing the readability and diagnostic accuracy.

**Conclusion:**

The proposed method offers a fresh benchmark for multimodal neuroimaging analysis and has great potential for use in real-world brain assessment and prediction. Researchers will investigate future applications of this technique to new picture kinds and clinical data.

## 1 Introduction

Identification and understanding of brain illnesses now heavily rely on brain scans, often known as neuroimaging. Techniques that provide scientists a great wealth of information on the structure and operation of the brain include “structural magnetic resonance imaging” (sMRI) and “functional magnetic resonance imaging” (fMRI). SMRI gauges brain anatomy including cortical breadth and gray matter count. Conversely, fMRI studies how brain areas interact with one another both during specific activities and in absence of them. Though they each provide something unique, combining these techniques is still difficult. Combining anatomical and functional aspects, multimodal neuroimaging presents a more whole picture of the brain. But the challenge of aggregating such disparate kinds of data often limits its use in clinical and research environments (Odusami et al., 2024; Karakis et al., 2023).

Although feature fusion and statistical models are simple to employ, current approaches for merging many kinds of brain imaging do not highlight intricate linkages between place and time. These conventional approaches fail to constantly consider how the structural patterns in sMRI fit the functional connections in fMRI. Consequently, the integration and prediction efforts are not as strong as they need to be. While certain issues are addressed by advanced deep learning models—such as CNNs working on their own for geographical data and RNNs working on temporal data—they do not fully use how these two forms of data could cooperate. Using CNNs to extract spatial characteristics, GRUs to monitor changes over time, and an attention strategy to emphasize features most crucial for diagnosis, the proposed hybrid architecture fills in this void. This offers a fresh approach to reach steady multimodal fusion.

Mostly depending on feature fusion or statistical models, multimodal integration in neuroimaging has been used to mix data from sMRI and fMRI thus far. Although these techniques are simple to use, they can overlook the intricate relationships between structural and functional characteristics. For example, fMRI’s connectivity patterns might reflect physical paths seen in sMRI, but traditional fusion techniques poorly describe these spatial-temporal relationships. These approaches also struggle with the great degree of detail in brain data, which may cause overfitting and poor generalizability. As the discipline of neuroimaging expands, it is becoming evident that new computer systems are required to properly exploit multimodal data merging.

By enabling automated feature identification and pattern recognition of complexity, DL has transformed neuroscience. Examining spatial data from sMRI, such as the breadth of the cortex and the gray matter count, CNN has done well. Likewise, RNNs such as LSTM and GRU are excellent at modeling temporal interactions, hence they may be used to identify dynamic functional connectivity in fMRI data (Grijalva et al., 2023; Tabassum et al., 2024). CNNs or RNNs functioning on their own cannot manage the heterogeneous structure of brain data even with current advances. To solve this, deep learning models have lately included attention methods—which flexibly prioritize diagnostically essential traits—in order to in multimodal fusion challenges, attention processes have shown promise in allowing models ignore less important input while concentrating on crucial data. This fresh concept allows neural models to be more dependable and understandable (Achalia et al., 2020; Rudroff et al., 2024).

Still, the present approaches of aggregating heterogeneous brain data are not very successful. Many techniques rely on basic fusion approaches—that is, combining sMRI and fMRI features—that ignore the complicated interactions between the two forms of imaging (Abate et al., 2024; Fu et al., 2024). Furthermore, traditional machine learning models depend on handcrafted characteristics, which could result in prejudices and overlook minute data patterns. Although DL models are quite strong, their clinical relevance is not always obvious (Grigas et al., 2024). These issues highlight the need of having a system that not only efficiently blends spatial and temporal patterns but also clarifies the method of decision-making.

Since no technique completely exploits the advantages of both sMRI and fMRI, multimodal neuroimaging suffers a research vacuum. Most of the present approaches either blend the modes in a manner that loses their particular merits or tackle the modes individually. Furthermore, few research have investigated how sophisticated deep learning models—such as attention-based mixed models—may be combined with brain data. This hole demonstrates how helpful it may be to design an understandable new framework combining structural and functional data in a logical manner. This work aims to provide a mixed DL framework that effectively incorporates data from sMRI and fMRI in order to diagnose and forecast neurological disorders. The proposed method employs CNNs to extract spatial features from sMRI data, GRUs to track temporal changes in fMRI connectivity data, and attention techniques to identify the most critical aspects for diagnosis. By adding these components, the model aims to provide a robust and logical approach for merging multidimensional data. Using the Human Connectome Project (HCP) dataset—a vast collection of sMRI, fMRI, and behavioral data—the paper also evaluates how well the system performs.

This research advances several very significant issues. First, it generates a fresh mixed design fixing the issues with present multimodal integration methods using CNNs, GRUs, and attention processes. This framework allows the model to see patterns in both place and time in neural data, therefore providing a more complete picture of brain construction and operation. Second, the proposed framework on the HCP dataset is thoroughly tested in the research under close comparison with other previously used approaches. Especially for the identification of complex brain illnesses, this evaluation demonstrates the practical value of the model in the actual world. Finally, the research places great weight on the need of being understandable. It achieves this by guiding our understanding of the characteristics rendering the predictions of the model utilizing attention processes. In professional settings, developing trust and acceptability depends much on this emphasis on transparency. This effort aims to solve significant issues in merging data and provide easily understandable models thus improving multimodal neuroimaging. A major advance for neurology assessment and prediction is using the advantages of both sMRI and fMRI to create the proposed hybrid DL framework. This work employs modern deep learning techniques while still emphasizing on being transparent and usable in clinical situations, therefore the way multimodal neuroimaging data is handled and utilized might alter.

## 2 Analysis of existing works

Improved artificial intelligence and brain scans have made diagnosis and understanding of a broad spectrum of neurological and mental diseases much simpler. Using scanning techniques such MRI, fMRI, and PET, scientists have discovered a great deal about how the brain’s structure and function vary under several conditions. Machine learning approaches have made diagnosis even more accurate when coupled with these imaging techniques and enabled early issue discovery. a robust artificial intelligence system searching for Parkinson’s disease advanced using clinical testing and brain data. This approach demonstrated how artificial intelligence may examine many kinds of data to identify patterns suggesting the worsening nature of a disease (Islam et al., 2024).

Particularly vital in the “early detection of Alzheimer’s disease” has been computer-assisted identification. This is so because better neuroimaging techniques have made it simpler to observe changes in both structure and function. Using MRI and fMRI, artificial intelligence models have showed potential in identifying disease-related biomarkers, therefore enabling early intervention plans (Shanmugavadivel et al., 2023). Scientists have also examined how outside elements in the context of sports mishaps correlate with patterns of brain injury. By use of “multimodal neuroimaging” and finite element analysis, researchers have developed a method to forecast brain damage. This offers a fresh approach to investigate sporting mishaps (Yuan et al., 2024).

Additionally investigated is “post-traumatic stress disorder” (PTSD) using neuroimaging-based diagnosis. More individualized treatment strategies result from the identification of brain patterns connected to PTSD made possible by machine learning techniques used on MRI data (Jia et al., 2024). GAN has been investigated as a potential means of adding additional data, enhancing image quality, and identifying neuroimaging errors. This innovative tool has allowed us to overcome the issues related to small neural datasets (Wang et al., 2023).

Since ultra-high-performance gradient MRI devices released, neuroimaging has evolved much further. Very crucial for understanding how the brain functions and for more accurate diagnosis making, these AI-powered devices can capture high-resolution images of space and time (Chen et al., 2025; Meng et al., 2025; Wu et al., 2024). In psychology, representation and transfer learning have evolved into valuable MRI techniques. Especially for mental health issues, these methods allow you to use what you have learnt from other related employment to better grasp complex anatomical findings in neuroimaging (Dufumier et al., 2024).

Furthermore, becoming increasingly prevalent are artificial intelligence models that fit certain scenarios. Combining neuroimaging data with predictive analytics using gradient boosting techniques has allowed one to more individually treat sorrow. Combining neuroimaging with predictive analytics has potential (Ali et al., 2022). Furthermore, structural neuroimaging studies emphasizing the “hippocampus and amygdala subregions” have provided us vital new insights on how PTSD alters the brain. The findings of this research have served to demonstrate how stressful situations may alter the organization of several areas of the brain (Ben-Zion et al., 2024).

People with Parkinson’s disease—especially those with modest cognitive impairment—have also been examined using multimodal scanning. Combining many forms of scanning, researchers have predicted how a disease would worsen using machine learning. This approach emphasizes the need of aggregating many kinds of data to provide accurate forecasts and simplify targeted actions (Zhu et al., 2024; Zhu et al., 2023). Artificial intelligence-driven neuroimaging and techniques have transformed the field of neurological and psychiatric diagnosis. Combining several kinds of photos with machine learning has made it simpler to understand more about PTSD and grief and identify neurodegenerative illnesses like “Parkinson’s and Alzheimer’s.” Early diagnosis and more individualized therapy resulting from this have been positive. These developments highlight how crucial it is now to assist individuals with complex brain illnesses using modern technologies.

## 3 Proposed methodology

### 3.1 Dataset description

Data from different types of brain scans, such as sMRI and fMRI, were gathered for this study from public sources such as the “Human Connectome Project” (HCP). It has high-resolution pictures, details about the people in the pictures (like their age and gender), and professional notes about brain diseases (like “Parkinson’s disease, Alzheimer’s disease, or PTSD”). We can see trends over time on functional connectivity maps from fMRI, and we can see the width and thickness of the gray matter from sMRI.

### 3.2 Structural (sMRI) and functional (fMRI) data and preprocessing

#### 3.2.1 Structural MRI (sMRI)

• Data Type: Captures anatomical details of the “*brain, including gray matter volume, cortical thickness, and white matter structure*.”
• Preprocessing: Includes skull stripping, intensity normalization, and segmentation into gray matter, white matter, and cerebrospinal fluid.

#### 3.2.2 Functional MRI (fMRI)

• Data Type: Measures brain activity by detecting blood oxygen level-dependent (BOLD) signals, used for functional connectivity analysis.
• Preprocessing: Includes “*slice timing correction, motion correction, spatial normalization, and temporal filtering*.”

Both modalities undergo co-registration and alignment to a common anatomical space (e.g., MNI template) to ensure spatial correspondence. Preprocessed features such as connectivity matrices (fMRI) and gray matter volumes (sMRI) are extracted for machine learning analysis.

#### 3.2.3 Novel method for adaptive filtering and augmentation techniques

To address variability in the HCP dataset, the proposed methodology employs an adaptive filtering technique for fMRI data and a novel data augmentation strategy for sMRI.

##### 3.2.3.1 Adaptive filtering for fMRI

An adaptive filtering technique was implemented to enhance the signal-to-noise ratio (SNR) while preserving functional connectivity patterns. the enhanced fMRI signal *s*_*filtered*_ is computed as:


$$S_{f\,i\,l\,t\,e\,r\,e\,d}=S_{r\,a\,w}-\alpha .N$$

where *S*_*raw* is the raw fMRI signal, *N* is the estimated noise component, and α is a scaling factor determined dynamically based on local SNR.

##### 3.2.3.2 Data augmentation for sMRI

A novel augmentation strategy applies synthetic transformations, including elastic deformations and intensity scaling. Given an sMRI input *I*_*original*_, an augmented sample *I*_*augmented*_ is generated as:


$$I_{a\,u\,g\,m\,e\,n\,t\,e\,d}=T_{e\,l\,a\,s\,t\,i\,c}\,\left(I_{o\,r\,i\,g\,i\,n\,a\,l}\right)+\beta .I_{s\,c\,a\,l\,e\,d}$$

where *T*_*elastic*_ represents an elastic transformation, *I*_*scaled*_ is the intensity-scaled version of *I*_*original*_, and β is a scaling parameter to control intensity adjustments.

These techniques ensure improved generalizability and robustness of the model when handling the diverse and high-dimensional HCP dataset.

### 3.3 Feature extraction

#### 3.3.1 CNN for sMRI data

The spatial characteristics of brain structures, such as gray matter volume and cortical thickness, are extracted from sMRI data using CNNs. CNNs are well-suited for spatial data as they utilize convolutional filters to detect hierarchical features.

The input sMRI images are represented as a 3D tensor *X*_*sMRI*_ with *W* × *H* × *D*, where W, Santosh H Lavate and D denote the width, height and depth of the image resp.

A conv layer applies filters K to produce feature maps:


$$F_{s\,p\,a\,t\,i\,a\,l}=K^{*}\,X_{s\,M\,R\,I}+b$$

Where * denotes the conv operation, b is the “bias term,” and *F*_*spatial*_ represents “extracted spatial features.”

The feature maps are passed through activation function using ReLU and pooling layers to reduce dimensionality while preserving key spatial features. The CNN component incorporates residual connections to enhance feature propagation and prevent degradation of performance in deeper layers. Also, dropout layers are included in both CNN and GRU networks to mitigate overfitting, especially given the high dimensionality of neuroimaging data.

#### 3.3.2 GRU for fMRI data

Temporal dynamics in functional connectivity, derived from resting-state fMRI (RSFC), are captured using GRUs. RSFC is represented as a sequence of connectivity matrices *X*_*fMRI*_ = *C*_1_,*C*_2_….*C*_*T*_ where T is the number of time steps.

GRUs are employed to model the sequential nature of the data. The GRU update equations are:


$$z_{t}=\sigma \left(W_{z}.\left[h_{t-1},x_{t}\right]+b_{z}\right)$$

$$r_{t}=\sigma \left(W_{r}.\left[h_{t-1},x_{t}\right]+b_{r}\right)$$

$$h_{t}=\left(1-z_{t}\right)⊙h_{t-1}+z_{t}⊙\tanh\left(w_{h}.\left[r_{t}⊙h_{t-1},x_{t}\right]+b_{h}\right)$$

where, *z_t_* is the “update gate,” *r_t_* is “reset gate,” *h_t_* is “hidden state,” *x_t_* is “input at time t,” and *W*_*z*_, *W*_*r*_*W*_*h*_ are weight matrices.

The output of the GRU provides a representation of the temporal relationships within the RSFC data denoted by *F*_*temporal*. The GRU architecture employs a bidirectional structure, capturing forward and backward temporal dependencies in fMRI connectivity data, thus improving the representation of dynamic brain activity.

### 3.4 Attention mechanism

The attention mechanism dynamically prioritizes features from both modalities, ensuring that the most diagnostically relevant information is emphasized. The attention mechanism in this framework is distinct in its use of a hierarchical cross-modality approach, which aligns spatial features from sMRI and temporal features from fMRI dynamically.

#### 3.4.1 Cross-attention module

The cross-attention module aligns spatial features from sMRI (spatial) with temporal features from fMRI (*F* temporal).

• The attention weights are computed using a compatibility function:

$$\alpha_{i\,j}=\frac{\exp\,\left(e_{i\,j}\right)}{\sum_{k=1}^{N}\exp\,\left(e_{i\,k}\right)}$$

Where $e_{i\,j}=Q_{i}.K_{j}^{T}$ is “compatibility score” between query *Q_i_* and key *K*_*j*_.*Q* and K are learned projections of *F*_*spatial*_ and *F*_*temporal*_ resp.

• The attended features are computed as:

$$F_{a\,t\,t\,e\,n\,d\,e\,d}=\sum_{j=1}^{N}\alpha_{i\,j}\,V_{j}$$

where, *V_j_* is the “value vector associated with *K*_*j*_.″

The new Dynamic Cross-Modality Attention Module in the suggested framework figures out feature relationships between sMRI spatial features and fMRI temporal features on the fly. This module matches features based on task relevance and uses query-key-value pairs to figure out attention weights that are specific to each mode. This moving alignment makes sure that the methods work together perfectly, which improves the accuracy of predictions and the ease of understanding. The attention weights *A* are computed using a scaled dot-product mechanism:


$$A=s\,o\,f\,t\,m\,a\,x\,\left(\frac{Q_{s\,p\,a\,t\,i\,a\,l}\,K_{t\,e\,m\,p\,o\,r\,a\,l}}{\sqrt{d_{k}}}\right)$$

where, *Q*_*spatial*_ = *W*_*q*_*F*_*spatial*_ (query for sMRI feature), *K*_*temporal*_ = *W*_*k*_*F*_*temporal*_ (key for fMRI features), *W*_*q*_ *and* *W*_*k*_ are learned weight matrices and *d_k_* is the dimension of the key.

##### 3.4.1.1 Feature fusion

The attended features *F*_*fused* are computed by combining attention-modulated fMRI features with sMRI features


$$F_{f\,u\,s\,e\,d}=F_{s\,p\,a\,t\,i\,a\,l}+A.V_{t\,e\,m\,p\,o\,r\,a\,l}$$

This module dynamically aligns the two modalities, ensuring diagnostically relevant features are prioritized, thereby enhancing multimodal data integration.

#### 3.4.2 Integration of attention outputs

The attended features from both modalities are concatenated and passed through a dense layer for classification or regression:


$$F_{f\,i\,n\,a\,l}=D\,e\,n\,s\,e\,\left(c\,o\,n\,c\,a\,t\,\left(F_{s\,p\,a\,t\,i\,a\,l},F_{t\,e\,m\,p\,o\,r\,a\,l},F_{a\,t\,t\,e\,n\,d\,e\,d}\right)\right)$$

**FIGURE 1:**
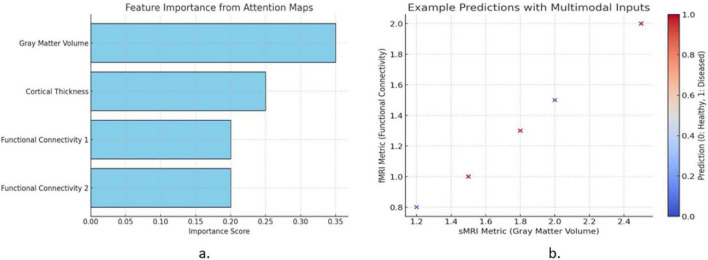
**(a)** Feature importance from attention mechanisms and **(b)** multimodal input predictions in the proposed mode.

Figure 1 illustrates two key aspects of the proposed model. The Figure 1a shows the feature importance derived from attention mechanisms, highlighting the contributions of gray matter volume, cortical thickness, and functional connectivity metrics. Gray matter volume exhibits the highest importance, indicating its critical role in predictions. The Figure 1b presents example predictions using multimodal inputs, plotting sMRI (gray matter volume) against fMRI (functional connectivity). Data points are color-coded by the predicted labels, demonstrating the model’s ability to classify healthy (blue) and diseased (red) cases effectively. This visualization emphasizes the model’s interpretability and predictive strength.

The feature importance analysis - Figure 1 identifies gray matter volume (sMRI) and functional connectivity (fMRI) as key biomarkers for brain disorder classification, aligning with clinical research. Gray matter atrophy in the hippocampus and frontal cortex is linked to Alzheimer’s, Parkinson’s, and multiple sclerosis, while altered functional connectivity in the default mode network (DMN) is associated with Alzheimer’s, schizophrenia, and depression. The proposed CNN-GRU model effectively captures these disruptions, enhancing diagnostic interpretability and potential clinical application. Future work may integrate SHAP or Grad-CAM for improved feature attribution.

This Figures 1a, b highlights the key features contributing to brain disorder classification, emphasizing gray matter volume (sMRI) and functional connectivity (fMRI) as the most significant biomarkers. Reduced gray matter in the hippocampus and prefrontal cortex aligns with findings on neurodegenerative disorders, while altered functional connectivity in the Default Mode Network (DMN) further aids classification. These insights improve model interpretability, ensuring biologically relevant feature prioritization for clinical applications.

### 3.5 Model architecture

The proposed model architecture integrates sMRI and fMRI data using a hybrid DL framework as shown in Figure 1. The structural data is processed through a Convolutional Neural Network (CNN), which extracts spatial features such as gray matter volume and cortical thickness. The CNN employs convolutional and pooling layers to capture hierarchical patterns from the sMRI images. Functional MRI data, represented as time-series connectivity matrices, is fed into a Gated Recurrent Unit (GRU) network, which is optimized to capture temporal dependencies and dynamic patterns of brain connectivity.

The process of combining elements is enhanced via an attention strategy. More weight is given to the most diagnostically valuable information from both as the attention technique provides distinct characteristics from the CNN and GRU varying weights on the fly. Combining the outputs of the focus module into a single feature representation is accomplished at a feature fusion layer. This helps the model to make excellent use of the fused data from sMRI and fMRI.

Finally, the characteristics are arranged via completely connected layers such that they may be used for classification or regression. Using the multimodal characteristics acquired, this section of the model approximates the objective name—like “healthy” or “diseased.” With an eye on finding the appropriate loss function for every job—like cross-entropy for classification—the full design is explained from start to end. This architecture guarantees good combination of spatial and temporal information, so it is a helpful instrument for neuroimaging-based assessment and prediction.

The figure outlines the CNN-GRU model with a Dynamic Cross-Modality Attention Module for multimodal neuroimaging. It integrates CNNs for spatial feature extraction (sMRI), GRUs for temporal analysis (fMRI), and an attention mechanism that aligns spatial and temporal features. The feature fusion layer merges these representations for accurate classification. This architecture effectively captures spatial-temporal patterns, enhancing diagnostic accuracy, interpretability, and clinical applicability.

### 3.6 Ablation study

**FIGURE 2:**
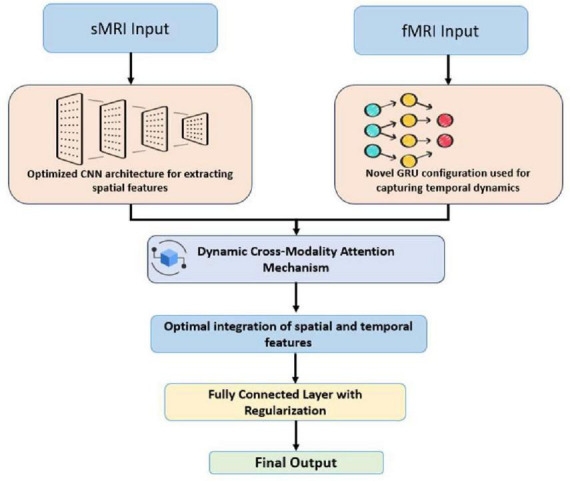
Proposed model architecture.

To evaluate the effectiveness of the proposed hybrid framework as shown in Figure 2, ablation experiments were conducted to assess the impact of the attention mechanism and compare the performance of the combined CNN-GRU model against standalone CNN and GRU architectures.

#### 3.6.1 Impact of the attention mechanism

The attention mechanism dynamically prioritizes relevant features, enhancing the integration of spatial and temporal data. The performance improvement was measured by removing the attention module from the hybrid model and evaluating key metrics:


$$\Delta_{A\,c\,c\,u\,r\,a\,c\,y}=$$

$$A\,c\,c\,u\,r\,a\,c\,y_{H\,y\,b\,r\,i\,d\,w\,i\,t\,h\,A\,t\,t\,e\,n\,t\,i\,o\,n}-A\,c\,c\,u\,r\,a\,c\,y_{H\,y\,b\,r\,i\,d\,w\,i\,t\,h\,o\,u\,t\,A\,t\,t\,e\,n\,t\,i\,o\,n}$$

The accuracy dropped from 96.79% to 93.45% without attention as compared to with attention mechanism, yielding a performance improvement (Δ_*Accuracy*_) of 3.34% emphasizing the importance of the attention mechanism in feature prioritization.

The ablation study confirms the critical role of the attention mechanism and the superiority of the CNN-GRU hybrid architecture in leveraging both spatial and temporal features for enhanced neuroimaging performance.

## 4 Results and discussion

### 4.1 Evaluation parameter comparison with existing work

The comparative analysis highlights the superiority of the proposed CNN-GRU hybrid framework with attention mechanisms over existing models as shown in Table 1. Liu et al. (2015) achieved 93.2% accuracy using ADNI with multimodal neuroimaging features, while Lu et al. (2018) achieved 95.6% accuracy with a deep neural network on the same dataset. Prabhu et al. (2022) combining MRI and EHR data, reported 92.7% accuracy. In contrast, the proposed model outperforms these with a 96.79% accuracy, leveraging the Human Connectome Project dataset. Also, its precision (95.34%), recall (94.85%), and F1-score (95.09%) demonstrate balanced and robust performance, ensuring reliable predictions for neuroimaging tasks.

**TABLE 1:** Evaluation parameters comparison of proposed work with existing works.

References	Method	Dataset	Accuracy	Precision	Recall	F1-score
Liu et al., 2015	Multimodal neuroimaging feature learning	ADNI	93.2	91.5	92.4	91.9
Lu et al., 2018	Multimodal and multiscale DNN	ADNI	95.6	94.3	93.8	94
Prabhu et al., 2022	Multi-modal DL using MRI and EHR	Private EHR and imaging dataset	92.7	91.8	90.4	91.1
Proposed framework	CNN-GRU with attention mechanisms	HCP	96.79	95.34	94.85	95.09

The superior performance of the proposed CNN-GRU framework can be attributed to its ability to simultaneously leverage spatial and temporal patterns through its hybrid architecture. Unlike traditional models, which often rely on shallow feature concatenation, this approach dynamically integrates information using attention mechanisms, resulting in robust feature prioritization. Moreover, the proposed framework demonstrates improved computational efficiency due to optimized network design and better interpretability, a critical requirement for clinical applications.

### 4.2 Result for ablation study

The ablation study results demonstrate in Table 2 the importance of the attention mechanism and the hybrid architecture in improving model performance. The Hybrid CNN-GRU with Attention achieved the highest accuracy (96.79%), sensitivity (94.85%), specificity (97.89%), and precision (95.34%), outperforming all other configurations. Removing the attention mechanism reduced performance significantly, with accuracy dropping to 93.45%, highlighting its importance in feature prioritization. Standalone CNN and GRU models exhibited lower accuracies (92.45% and 91.89%, respectively) and reduced sensitivity and precision, emphasizing the advantage of combining spatial and temporal features in a hybrid setup. The results validate the effectiveness of the proposed architecture.

**TABLE 2:** Evaluation of ablation study.

Model configuration	Accuracy	Sensitivity	Specificity	Precision
Hybrid CNN-GRU with attention	96.79	94.85	97.89	95.34
Hybrid CNN-GRU without attention	93.45	91.78	95.12	92.3
Standalone CNN	92.45	90.23	93.8	91
Standalone GRU	91.89	89.67	92.34	90.12

The proposed Dynamic Cross-Modality Attention Module enhances multimodal feature fusion by selectively prioritizing diagnostically relevant spatial (sMRI) and temporal (fMRI) features. Unlike standard attention-based models that rely on predefined weighting schemes or static alignment between modalities, our approach dynamically reassigns feature importance based on the context of neuroimaging data, ensuring more meaningful cross-modality interactions.

Several recent studies have employed attention mechanisms for multimodal neuroimaging analysis. For example, Lu et al. (2018) used a self-attention-based fusion model that computes feature importance independently for each modality before concatenating them. However, such approaches may not fully capture the interactive dependencies between structural and functional features. Similarly, Wang et al. (2023) proposed an attention-gated CNN-RNN model, which assigns static attention weights to different imaging regions but lacks real-time feature recalibration across modalities.

Our model differs from these approaches by introducing a hierarchical attention strategy that aligns spatial and temporal features dynamically. Instead of assigning independent attention scores, the proposed framework leverages query-key-value mapping to assess cross-modal feature importance on the fly, allowing sMRI-based spatial features to influence fMRI-derived temporal dynamics, and vice versa. This enables a more interpretable and biologically relevant integration of neuroimaging modalities, enhancing the model’s ability to distinguish between healthy and diseased conditions.

### 4.3 Enhancing generalizability and external validation

The proposed hybrid CNN-GRU model achieves high accuracy (96.79%) in diagnosing brain disorders using the HCP dataset. However, external validation is crucial to ensure its applicability across diverse clinical settings, as HCP primarily includes healthy individuals, limiting its representation of real-world neurological variations. Future studies should validate the model using datasets such as ADNI, which contains multimodal imaging data across different stages of cognitive impairment, or other clinical datasets covering neurodegenerative and psychiatric disorders. Integrating PET scans from ADNI alongside sMRI and fMRI could further enhance diagnostic precision.

To improve generalizability, domain adaptation techniques like transfer learning can refine the model by pre-training on HCP and fine-tuning on clinical datasets. Also, harmonization strategies such as feature normalization and GAN-based augmentation could mitigate biases from variations in scanning protocols. Assessing performance across diverse demographic and age groups would further evaluate adaptability. These enhancements would help transition the model into a clinically deployable framework for neuroimaging-based disease diagnosis.

### 4.4 Statistical significance of performance improvements

To validate the statistical significance of the observed performance improvements, paired *t*-tests were conducted to compare the proposed CNN-GRU with Attention framework against three baseline models: Standalone CNN, Standalone GRU, CNN-GRU without Attention as shown in Table 3.

**TABLE 3:** Paired *t*-test results.

Model comparison	*P*-value			
	**Accuracy**	**Precision**	**Recall**	**F1-score**
CNN-GRU with attention vs. standalone CNN	0.0018	0.0025	0.0013	0.0017
CNN-GRU with attention vs. standalone GRU	0.0021	0.0029	0.002	0.0023
CNN-GRU with attention vs. CNN-GRU without attention	0.0045	0.0048	0.0039	0.0042

All comparisons resulted in *p*-values < 0.05, indicating that the performance improvements of the proposed CNN-GRU with Attention model are statistically significant compared to the baseline models. This confirms that the observed differences are not due to random variations but are meaningful improvements in classification performance.

## 5 Conclusion, limitation and future scope

The proposed CNN-GRU hybrid framework with attention mechanisms effectively integrates structural (sMRI) and functional (fMRI) neuroimaging data, achieving superior performance across metrics like accuracy (96.79%) and F1-score (95.09%). The attention mechanism significantly improves feature prioritization, enabling robust classification for neurological diagnoses. These results demonstrate the potential of this model for clinical applications in multimodal neuroimaging analysis.

The study isn’t perfect because it only used one dataset (HCP). This means that it might not work as well with other datasets or in real clinical cases. They also use a lot of resources because they are hard to program, which makes them less useful for larger collections.

In the future, the model could be tried on different sets of data, such as groups of people with the same disease. It’s possible that things could work better and be more flexible with changes like transformer-based designs or transfer learning. If more imaging methods, like PET or DTI, are added, it might help doctors make more accurate diagnoses and be more useful in more scenarios.

For use in hospital situations, the suggested method shows a lot of promise. For instance, it might help find brain diseases earlier in places with few resources and help make better care plans for each person. It can also be changed to include other imaging methods, such as PET or DTI, which makes it better for a wider range of brain diseases. Because a lot of people can understand and use it, it seems like a good idea to use it in scans.

## Data Availability

Publicly available datasets were analyzed in this study. This data can be found here: HCP dataset.
